# Identification of key genes involved in the biosynthesis of triterpenic acids in the mint family

**DOI:** 10.1038/s41598-019-52090-z

**Published:** 2019-11-01

**Authors:** Zahra Aminfar, Babak Rabiei, Masoud Tohidfar, Mohammad Hossein Mirjalili

**Affiliations:** 1 Department of Agronomy and Plant Breeding, Faculty of Agricultural science, University of Guilan, Rasht, Iran; 2 Department of Plant Biotechnology, Faculty of Sciences & Biotechnology, Shahid Beheshti University G.C., Tehran, Iran; 3 Department of Agriculture, Medicinal Plants and Drugs Research Institute, Shahid Beheshti University, G. C., Tehran, Iran

**Keywords:** Molecular engineering in plants, Plant molecular biology

## Abstract

Triterpenic acids (TAs), a large group of natural compounds with diverse biological activity, are produced by several plant taxa. Betulinic, oleanolic, and ursolic acids are the most medicinally important TAs and are mainly found in plants of the mint family. Metabolic engineering is strongly dependent on identifying the key genes in biosynthetic pathways toward the products of interest. In this study, gene expression tracking was performed by transcriptome mining, co-expression network analysis, and tissue-specific metabolite-expression analysis in order to identify possible key genes involved in TAs biosynthetic pathways. To this end, taxa-specific degenerate primers of six important genes were designed using an effective method based on the MEME algorithm in a phylogenetically related group of sequences and successfully applied in three members of the Lamiaceae (*Rosmarinus officinalis*, *Salvia officinalis*, and *Thymus persicus*). Based on the results of in-depth data analysis, genes encoding squalene epoxidase and oxido squalene cyclases are proposed as targets for boosting triterpene production. The results emphasize the importance of identifying key genes in triterpene biosynthesis, which may facilitate genetic manipulation or overexpression of target genes.

## Introduction

Terpenoids are a group of natural compounds derived from five-carbon isoprene units and are classified according to the number and structural organization of these biosynthetic building blocks. Among the different structural categories (mono-, sesqui-, di-, tri, tetra, and polyterpenoids), the largest and most diverse group are the triterpenoids (TTs, C_30_H_48_), with a wide distribution in plants^1^. These organic compounds can be further subdivided into subclasses based on the number of available rings in the structure. Three well-known triterpenic acids (TAs) of the lupane, oleanane and ursane type, namely betulinic acid (3β-hydroxylup-20(29)-en-28-oic acid, BA), oleanolic acid (3β-hydroxyolean-12-en-28-oic acid, OA), and ursolic acid (3β-hydroxyurs-12-en-28-oic acid, UA), have been frequently reported from different plant organs such as leaves, flowers, berries, and fruits^2–4^. Various biological effects of TAs including anti-inflammatory and antioxidant^5^, anti-HIV^6^, anti-fungal and immunomodulatory activities^7,8^ have been characterized. Additionally, extensive bioassays of TAs against different human cancer cell lines have been performed^9–16^. Recently, potential effects of BA, OA, and UA for the treatment of type 2 diabetes (T2DM) have been highlighted^17^. Therefore, due to this considerable pharmacological activity and potential high demand for these medicinally important compounds, there is an urgent need to find the best natural source and biotechnological approach for their production. TAs have been reported from a range of plant families including Dilleniaceae^18^, Ericaceae^19,20^, Euphorbiaceae^21^, Rosaceae^22^ and Myrtaceae^23^. The mint family (Lamiaceae) is considered to be a particularly rich source of TAs^24^, which have been isolated and quantitatively determined in *Salvia* species^25–27^, *Rosmarinus officinalis*^26^, *Thymus* species^28^, *Prunella vulgaris*^29^, *Orthosiphon stamineus*^30^, and *Melissa officinalis*^24^, among others.

**Figure 1:**
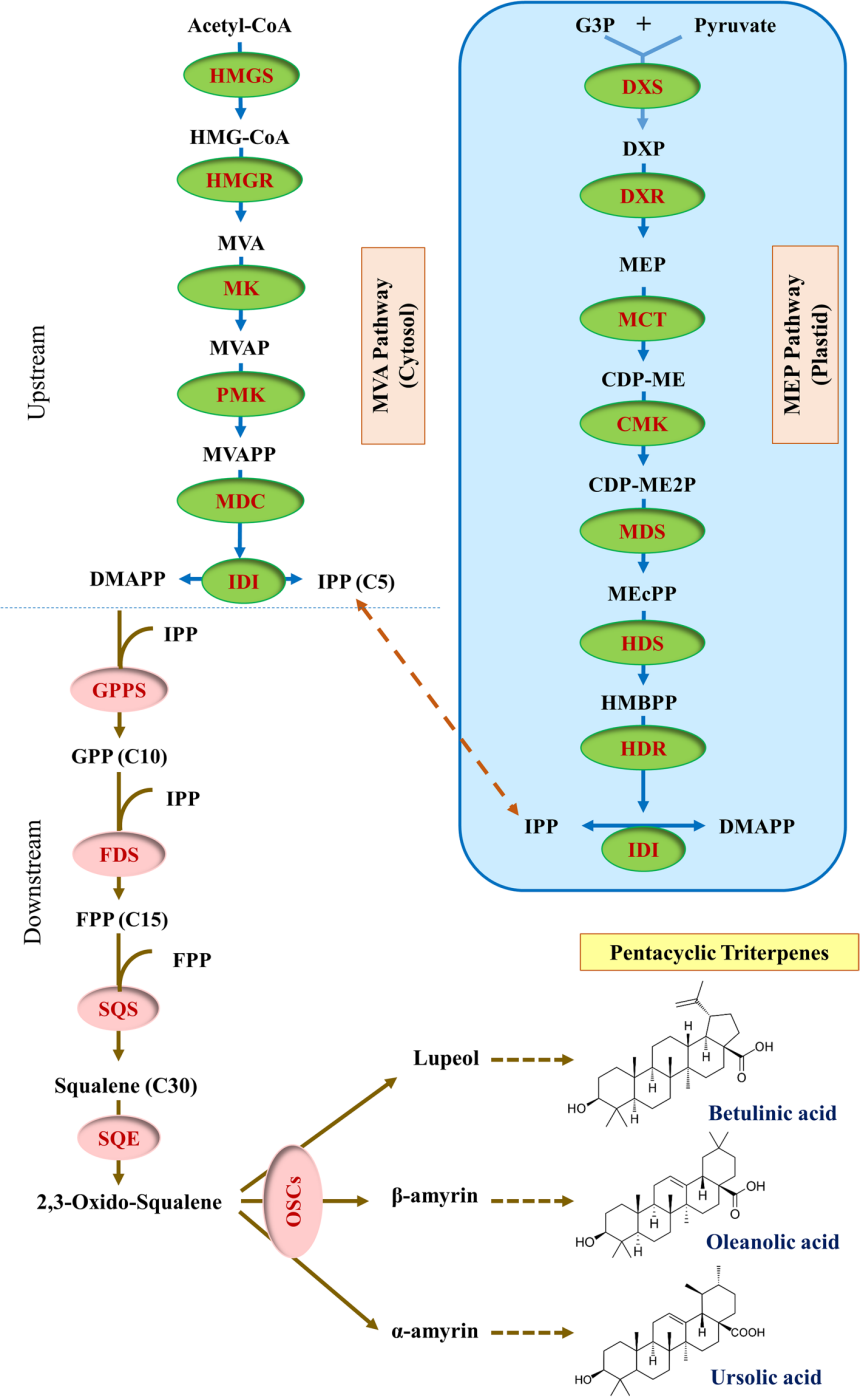
The biosynthetic route to pentacyclic triterpenes of betulinic, oleanolic and ursolic acids. These compounds are synthesized via the mevalonic acid (MVA) and methylerythritol 4-phosphate (MEP) pathway. The MVA pathway enzymes are shown on the left and the MEP pathway enzymes on the right in a blue box. The enzymes that catalyze the various steps are indicated in ovals. Enzyme abbreviations: DXS, 1-deoxy-d-xylulose-5-phosphate synthase; DXR, 1-deoxy-d-xylulose-5-phosphate reductoisomerase; MCT, 2-C-methyl-d-erythritol 4-phosphate cytidylyltransferase; CMK, 4-(cytidine 5′-diphospho)-2-C-methyl-d-erythritol kinase; MDS, 2-C-methyl-d-erythritol 2,4-cyclodiphosphate synthase; HDS, 4-hydroxy-3-methylbut-2-enyl diphosphate synthase; HDR, 4-hydroxy-3-methylbut-2-enyl diphosphate reductase; HMGS, 3-hydroxy-3-methylglutaryl coenzyme A synthase; HMGR, 3-hydroxy-3-methylglutaryl coenzyme a reductase; MK, Mevalonate kinase; PMK, phosphomevalonate kinase; MDC, mevalonate-5-decarboxylase; IDI, isopentenyl-diphosphate isomerase; GPPS, geranyl diphosphate synthase; FDS, farnesyl pyrophosphate synthase; SQS, squalene synthase; SQE, squalene monooxygenase or epoxidase; OSCs, oxido squalene cyclases.

Plant isoprenoids are derived from the cytoplasmic mevalonate (MVA) and the plastidial methylerythritol phosphate (MEP) pathways, which generate the isoprenoid precursors dimethylallyl pyrophosphate (DMAPP) and isopentenyl pyrophosphate (IPP). TAs are biosynthetically produced through the cyclization of squalene^31^, a hydrocarbon reported to be the precursor of all isoprenoids in eukaryotes. After the conversion of squalene to 2,3-oxidosqualene, cyclization by specific oxidosqualene cyclases (OSCs) generates a wide variety of triterpenoid backbones, including lupeol, β-amyrin, and α-amyrin, which are decorated by multiple cytochrome P450-dependent monooxygenases (P450s) to produce BA, OA and UA, respectively^32,33^. The main steps of TAs biosynthesis are shown in Fig. 1.

A large number of studies have shown a strong link between enzyme expression and metabolite production^34–37^, although the role of other regulatory mechanisms, including transcriptional, translational and post-translational regulation, is still unclear. In addition, the whole transcriptome should be considered, as the final product of a metabolic pathway is determined by a series of gene actions. High-throughput transcriptomics produces extensive transcript datasets that can be applied to identify candidate key genes in particular processes using co-expression networks analysis^38,39^. Examples include the identification of genes involved in disease^40^, cancer^41–43^, pathogen resistance^39,44,45^, different organs or developmental stages^46–48^ and biotic and abiotic stress in plants^49–51^. After the crucial genes are identified, the sequences encoding TAs biosynthetic enzymes need to be elucidated for molecular applications such as metabolic engineering. In the candidate gene approach, genes exhibiting a significant sequence similarity, involved in the same biological process so they can amplify by well-designed degenerate primers in the related organisms^52^. Targeted sequencing by these primers reduces the cost and time of sequencing projects and enables a more in-depth evaluation of key biosynthetic genes. Successful amplification requires the design of universal degenerate primers for members of a particular plant family, an example being the amplification of a key gene encoding enzymes involved in proanthocyanidins (PAs) biosynthesis in the Rosaceae family^53^.

There is a lack of genomic data for non-model plants, especially medicinal plants, but *in silico* expression analysis can bridge the knowledge gap and facilitate the identification of the main genes affecting the production of secondary metabolites (SMs). In the present study, the expression profiles of mRNA related to different organs in plants in the mint family under elicitation were analyzed for *in silico* studies. Co-expression network analysis was used to identify the key genes and their expression pattern in the TAs biosynthetic pathway. The genes were then amplified using designed degenerate primers in selected members of the mint family. Finally, biosynthetic genes related to BA, OA and UA production were analyzed by real-time polymerase chain reaction (RT-PCR), and high-performance liquid chromatography (HPLC) was performed to check whether the transcript levels were directly related to the TAs content. The results provide valuable information for biotechnological applications aimed at improving TAs production in plants of the mint family.

## Results

### Highlighting the expression pattern of TAs biosynthetic genes from high-throughput expression data

Transcriptome analysis experiments provide researchers with a snapshot of a subset of the expressed target genes under various conditions. The current study focused on the expression pattern of TAs biosynthetic genes through analyzing the gene expression profiles of Lamiaceae plants obtained from high-throughput transcriptome profiling experiments. In order to gain more insight into the TAs biosynthetic pathway, expression data sets were analyzed, and differentially expressed genes (DEGs) were identified using the BLAST algorithm, and a co-expression network was constructed to distinguish the key genes in the pathway. The retrieved expression data of TAs genes were analyzed, clustered based on the similarity of their expression pattern, and visualized by R software.

**Figure 2:**
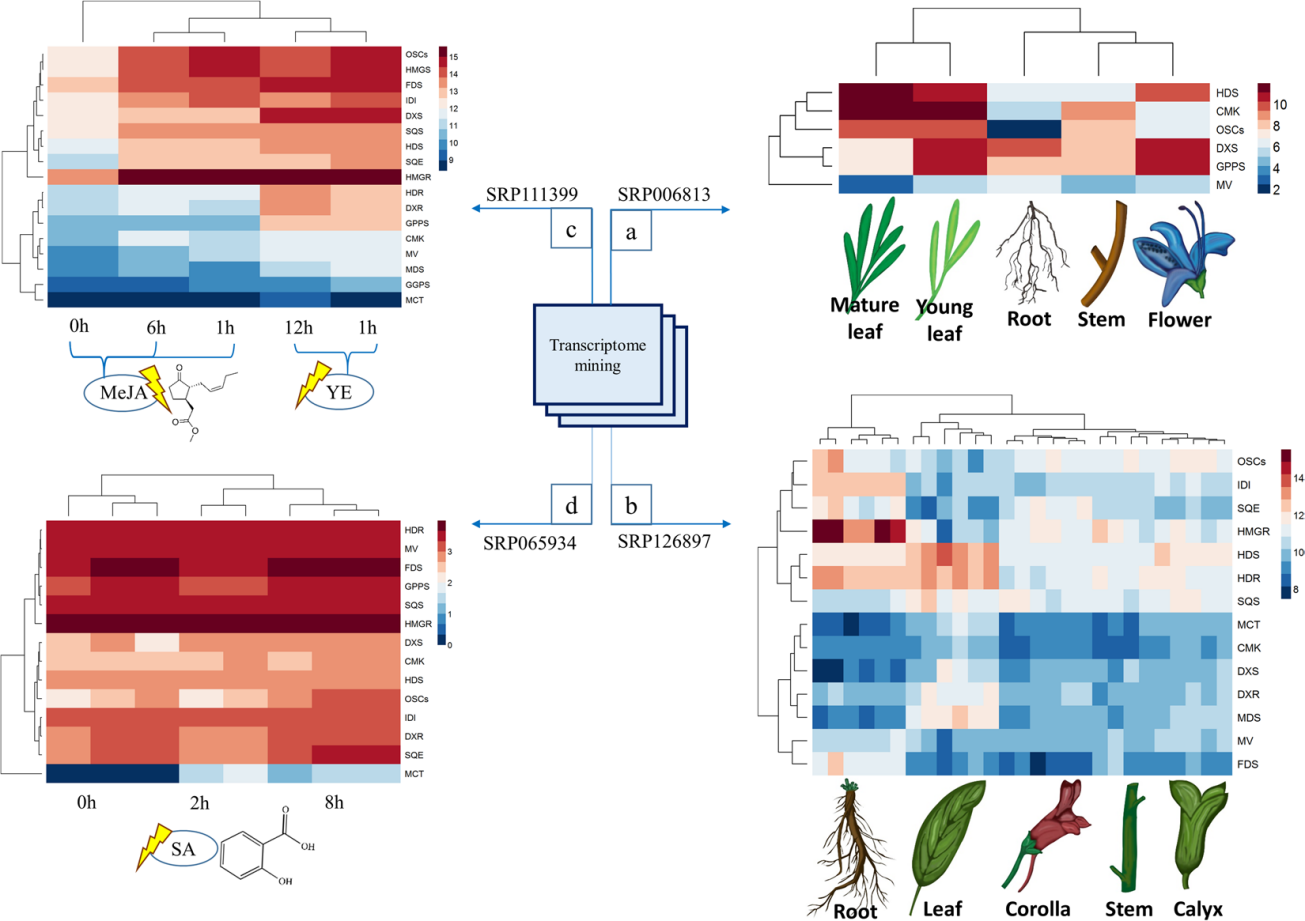
Heat map showing the expression profiles of TAs genes in four series of high-throughput sequencing data. (**a**) SRP006813- RNA sequencing of *Rosmarinus officinalis* (different tissues), (**b**) SRP126897- RNA sequencing of *Salvia splendens* (different tissues), (**c**) SRP111399- Transcriptome profiling of *Salvia miltiorrhiza* (elicited by methyl jasmonate (MeJA) and yeast extract (YE)) (**d**) SRP065934- Transcriptome profiling of *Salvia miltiorrhiza* (elicited by salicylic acid (SA)). Transcripts for some genes involved in TAs biosynthesis were not detected in experiment 1 because of the low size of reads, and so these genes were not included.

**Figure 3:**
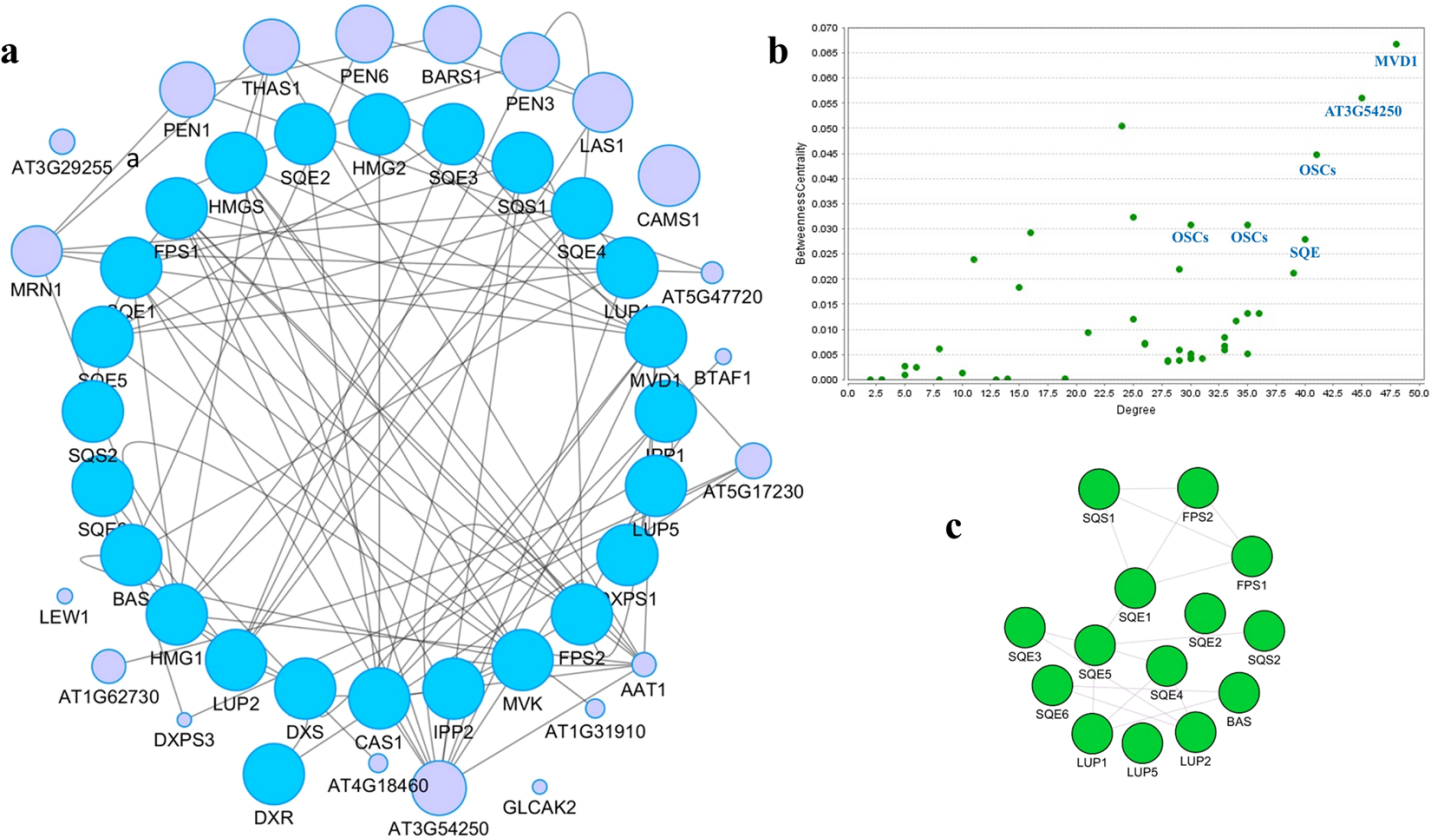
(**a**) Co-expression network constructed by geneMANIA based on the TAs biosynthetic genes in *A. thaliana*. (**b**) Scatter plot of degree and betweenness values for all nodes. (**c**) Co-expression relationships of downstream genes of TAs biosynthesis.

As shown in Fig. 2, a relationship was observed between the gene expression values and the specific tissue/elicitor. In general, biosynthetic genes in plants show a spatio-temporal expression pattern (particular tissue /developmental stage). In addition, these genes can be induced by stressful conditions or by elicitors such as MeJA^54–56^. Hierarchical clustering of gene expression values revealed two main clusters (Fig. 2b,c). If the genes involved in TAs biosynthesis are divided into upstream (MVA and MEP pathway) and downstream (*FDS*, *SQS*, *SQE*, *OSCs*), a downstream biosynthetic gene cluster is apparent, and most of the genes are overexpressed under elicitation compared with upstream genes (Fig. 2c). The co-expression network of upstream and downstream TAs biosynthetic genes was analyzed with network parameters, as illustrated in Fig. 3. Nodes are considered as the genes, and edges indicate the magnitude of their co-expression. A scatter-plot of the betweenness centrality (BC) versus degree (Fig. 3b) was generated to identify genes with high relative values in both parameters. *MVD1* (mevalonate diphosphate decarboxylase, *MDC* in Fig. 1), *AT3G54250* (GHMP kinase family proteins), *OSCs* and *SQE* showed high values based on these two parameters. Further, these nodes ranked highly according to other centrality parameters of closeness, stress, and radiality. However, when considering eigenvector parameters, the *OSCs* and *SQE* transformed the top ranking. The eigenvector centrality measures the influence of a node on the network. *Arabidopsis MVD* is encoded by two loci, and is responsible for the last step of the MVA pathway where mevalonic acid is converted into isopentenyl diphosphate (IPP). Regarding the co-expression relationship of downstream genes with the TAs pathway, *SQE* co-expresses with different triterpene cyclases (*OSCs*). Triterpene cyclases use 2,3-oxidosqualene as a substrate, a common biosynthetic intermediate for plant triterpenes, and convert it into a variety of pentacyclic triterpene scaffolds. *OSCs* can be multifunctional or produce only one product. Lupane, oleanane, and ursane skeletons are catalyzed by lupeol synthase (*LUS*), β-amyrin synthase (*BAS*), and α-amyrin (*AAS*) synthase or mixed-function amyrin synthase (*MFAS*) as *OCSs*, respectively. The pentacyclic skeletons are usually modified further to produce BA, OA and UA by cytochromes P450, acetyltransferases and glycosyltransferases^57^.

### Bioinformatics analysis to design degenerate primers and RT-PCR

**Figure 4:**
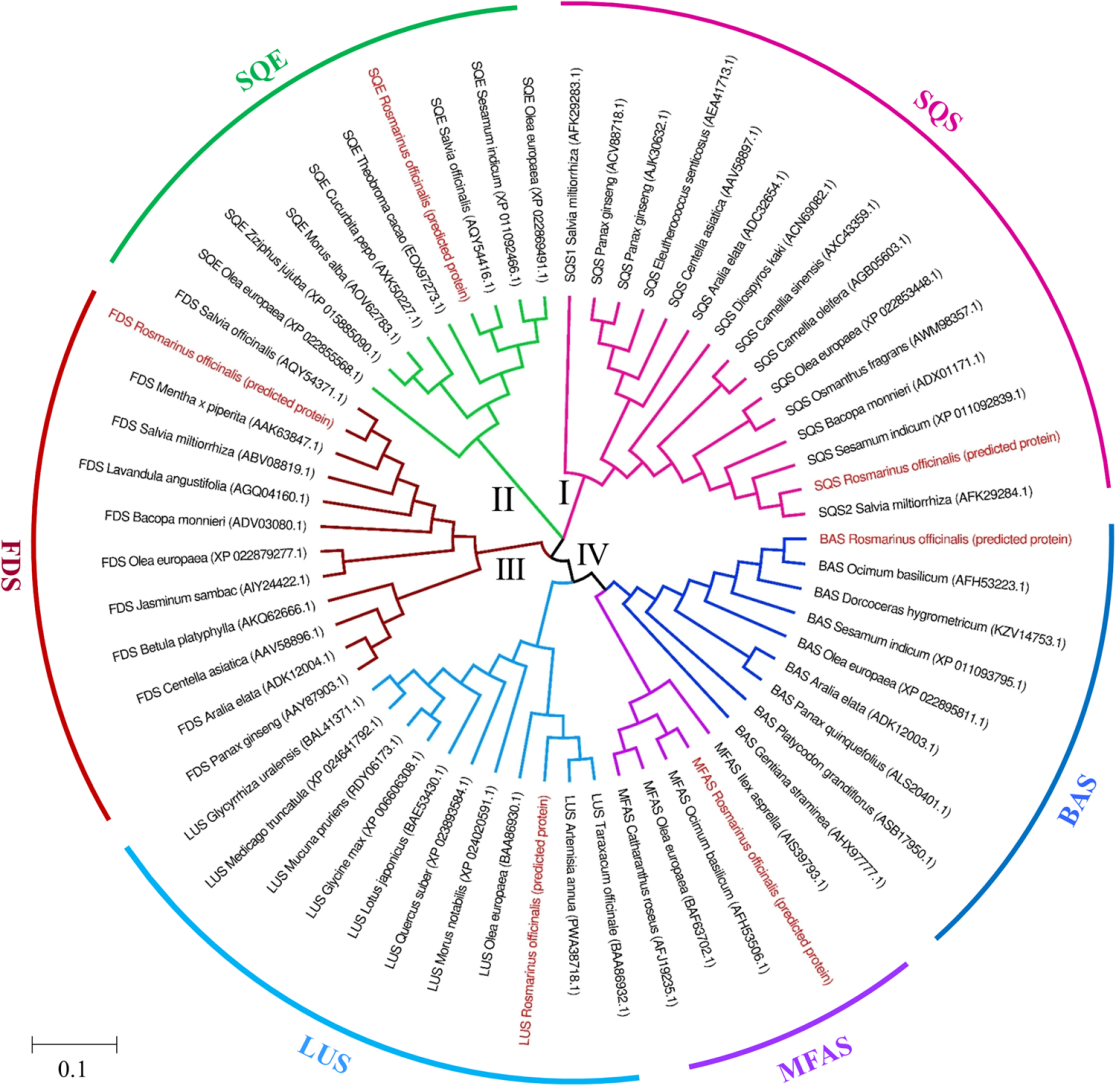
Phylogenetic analysis of TAs biosynthesis enzymes. The amino acid sequences were retrieved from GenBank (http://www.ncbi.nlm.nih.gov/genbank/). Predicted proteins of *R. officinalis* are shown in red. The tree was constructed using MEGA 7.0 software with an evaluation method of Bootstrap repeated a 1000 times. The scale bar represents the number of amino acid substitutions per site.

RNA-Seq provides a unique combination of transcriptome which can be considered as a repository for selecting the targets of interest. To design degenerate specific taxa primers for validating the expression patterns of TAs genes, nucleotide sequences of *FDS*, *SQS*, *SQE*, and *OSCs* (*LUP*, *BAS*, and *MFAS*) were retrieved from the transcriptome of rosemary (SRP006813). The predicted amino acid sequences were then subjected to phylogenetic analysis and the results (Fig. 4) indicate that all proteins of the TAs pathway can be divided into four large groups (I, II, III, and IV). Among these groups, branch IV consisted of OSCs and was further divided into three subgroups (LUS, BAS, and MFAS). These enzymes exhibited a much closer evolutionary relationship with each other. The reaction pathways by which TAs are generated via oxidosqualene cyclization catalyzed by the OSC family are divergent in nature. Members of this enzyme family have a very high sequence identity, which poses a challenge for determining exactly how each OSC controls the product specificity. However, some OSCs are multifunctional and normally convert 2,3-oxidosqualene into a variety of products. For example, MFAS cyclizes 2,3-oxidosqualene to α-amyrin and β-amyrin in a 5:3 ratio, which was demonstrated in experiments in yeast in which overexpression of MFAS in *Ocimum basilicum* led to the preferential production of α-amyrin^56^. Based on the sequence analysis, *BAS* and *MFAS* in this plant shared 73% identity, indicating that only a slight difference in their sequences may cause differences in the catalytic functions of OSCs.

**Figure 5:**
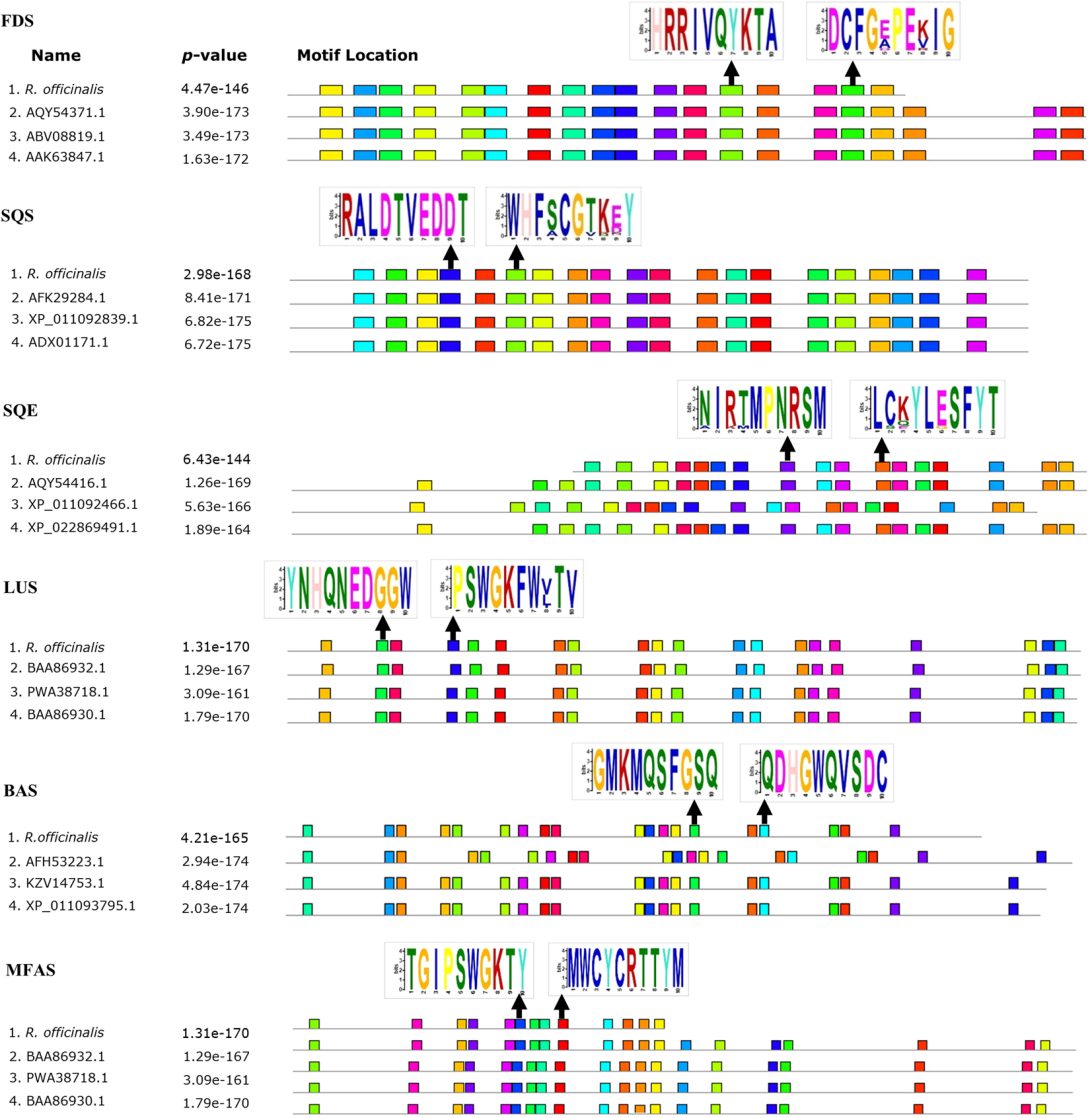
MEME general results. Every colored box represents a specific motif. The amino acid sequences of motifs used to design primers are shown with an arrow. Due to the limited space, only four sequences of each protein are shown.

Predicted proteins of LUS, BAS, and MFAS of *R. officinalis* were more phylogenetically related to LUS from *Taraxacum officinalis* and *Artemisia annua*, and BAS and MFAS from *O. basilicum*, sharing 83%, 78%, 88%, and 90% identity at the amino acid sequence level, respectively. As depicted in Fig. 4, the branching pattern of the phylogenetic tree reflected that the predicted amino acid sequences of FDS, SQS, and SQE were phylogenetically related to these enzymes in *Salvia*, the most closely related species to *R. officinalis* in the mint family, with the highest sequence identity (96%), and evolving from a common ancestor. The amino acid sequences of species closely related to *R. officinalis* according to the phylogeny tree were aligned, and the conserved blocks of sequences were identified by the Multiple Expectation – Maximization for Motif Elicitation (MEME) algorithm. Figure 5 displays the motif pairs used to design the primer pairs for each gene. Table 1 indicates the designed degenerate primers that passed the specificity checking process.

**Table 1:** Designed degenerate primers specific for triterpenic acid biosynthetic genes in the members of the Lamiaceae used in this study.

Gene name	Primer	Primer sequence (5′ → 3′)	Amplicon size
*FDS*	FDSFwd	CATCGCCGCATTGTWCAG	187
	FDSRev	CAATCTTTTCRGGCTCACC	
*SQS*	SQSFwd	GCTTGACACWGTTGAGGA	111
	SQSRev	GTACCRCATGAAAAATGCCA	
*SQE*	SQEFwd	GACCATGCCMAACAGAAG	209
	SQERev	TGTAGAAGGAYTCAAGATACT	
*LUS*	LUSFwd	ATCAGAAYGAAGATGGAGG	200
	LUSRev	CCARAACTTTCCCCACGA	
*BAS*	BASFwd	GGAATGAAGATGCAGAGYTT	227
	BASRev	TGCCATCCATGATCTTGRTC	
*MFAS*	MFASFwd	TCNTGGGGAAAGACNTAT	131
	MFASRev	CGGCARTAACACCACAT	

The position of degeneracies in the oligonucleotides are shown in the bold IUPAC ambiguity codes.

**Figure 6:**
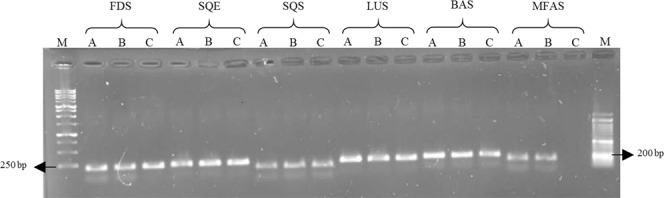
The results of RT-PCR for TAs biosynthetic genes in *R. officinalis* (**A**), *S. officinalis* (**B**), and *T. persicus* (**C**). The first and the last lane (M) are 1 kb and 50 bp markers, respectively. Amplification products were separated on a 1.5% agarose gel.

In order to illustrate the performance of the designed primer in amplifying target fragments of the TAs biosynthetic genes from Lamiaceae plants, a two-step RT-PCR was performed and gel electrophoresis was used to visualize the results (Fig. 6). Based on the results, a single DNA product matched the expected size of the PCR amplicon for each gene in three members of the Lamiaceae family, which indicated that the primers worked efficiently amplified the amplicons produced by degenerating specific taxa primers. However, *MFAS* primers failed to amplify the responding gene in *T. persicus* and did not show any band at all, maybe because the sequence of this gene was available only in a few plants and it was not sufficient for designing universal primers.

Subsequently, the amplicons were purified, sequenced, and then analyzed by bioinformatics tools to confirm the results of the RT-PCR analysis. In addition, all of the sequences were successfully affiliated and corresponded to the target genes of the TAs biosynthetic pathway according to the BLAST sequence analysis.

### Tissue-specific gene expression and TAs Profile

**Figure 7:**
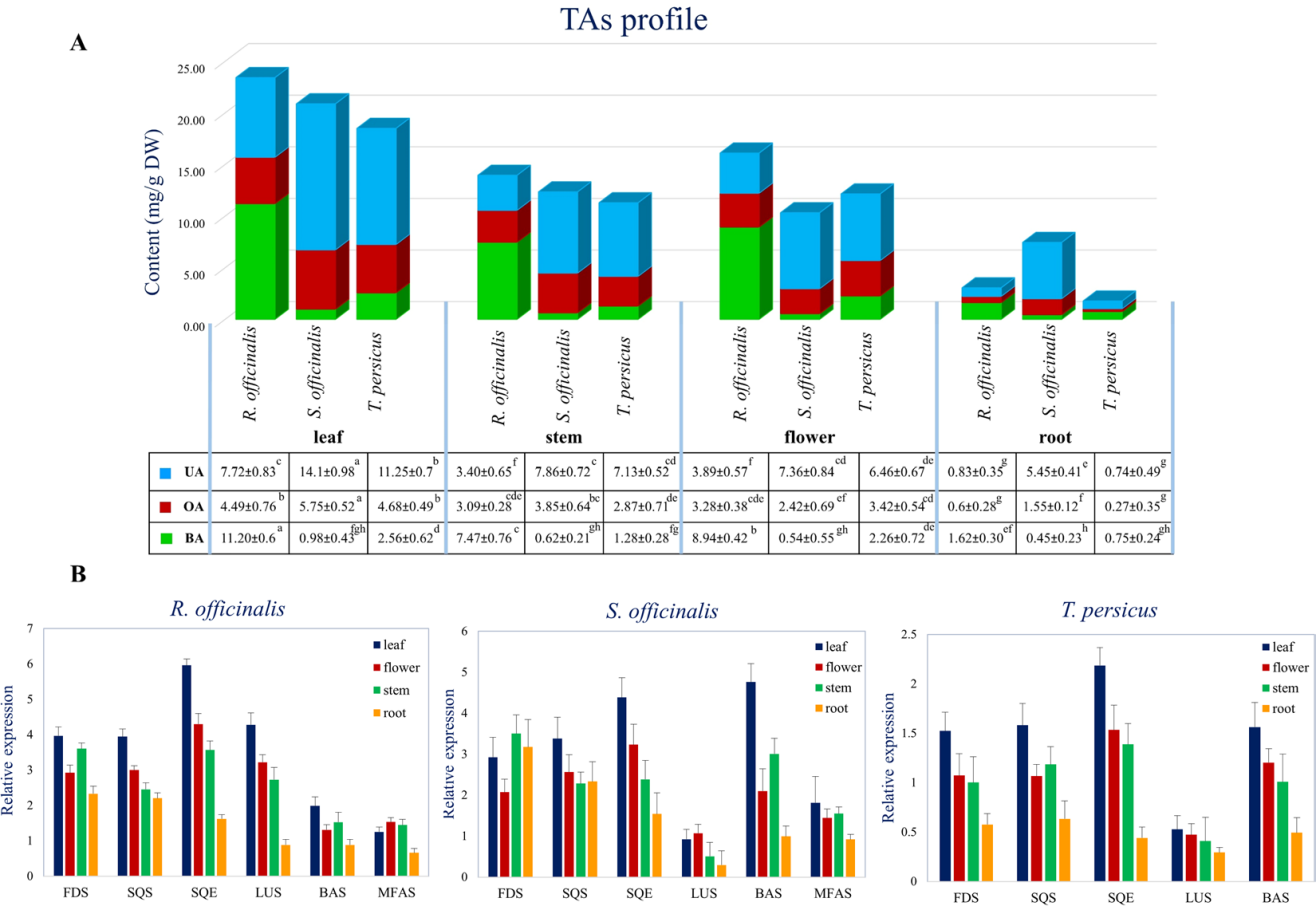
Tissue-specific changes in TAs biosynthesis in metabolite and transcript levels. (**a**) Estimation of TAs contents (mg/g DW) in different tissues of three members of the Lamiaceae family. The bars represent total measured TAs and the panel are means ± SD. Significance levels for the difference between any pair of means (TAs content) by Duncan’s multiple range test are marked above the data in lowercase. (**b**) Transcript abundance of TAs biosynthetic genes, relative transcript levels of farnesyl pyrophosphate synthase (*FDS*), squalene synthase (*SQS*), squalene monooxygenase or epoxidase (*SQE*), beta-amyrin synthase (*BAS*), and mixed function amyrin synthase (*MFAS*) genes were determined by qRT-PCR in different plant organs of *R. officinalis*, *S. officinalis* and *T. persicus*. The mRNA levels were normalized with reference to the housekeeping gene *GAPDH*. Error bars represent standard deviation from the mean (n = 3).

In order to determine the tissue-dependent expression of TAs enzymes, a panel of 6 genes, *FDS*, *SQS*, *SQE*, *LUS*, *BAS*, and *MFAS*, were profiled in four tissues: leaf, flower, stem, and root. The average contents of BA, OA, and UA in different tissues of *R. officinalis*, *S. officinalis*, and *T. persicus* were determined using the standard equations and expressed in mg/g dry weight (DW) of the material (Fig. 7a). Although a large body of phytochemical research on TAs evaluation has been quantitatively conducted in plants, including different species of the Lamiaceae family^26,27^, no studies have focused on the TAs profile in different tissues of these plants except *Salvia*^58^. Tissue-specific gene expression and TAs profile is reported in the present study for the first time.

Based on the results obtained by ANOVA, BA, OA, UA and their total (TAs) content (mg/g DW) were statistically significant (P < 0.05) in different tissues and plant species, and in their interactions. In the plant species studied, the highest and the lowest content of TAs was observed in the leaf and root, respectively (Fig. 7a). *R. officinalis* showed the highest TAs concentration in all tissues except the root, while *S. officinalis* contained a higher content of TAs (7.50 ± 0.16) in the root in comparison with *R. officinalis* and *T. persicus*.

These results are consistent with those of an earlier report of TAs distribution in various plants, which introduced the Lamiaceae family as a particularly rich source of BA, OA, and UA, with the highest concentration measured in rosemary leaves^24,26^.

Regarding the studied TAs, the maximum content of BA was quantified in leaves of *R. officinalis* (11.20 ± 0.6 mg/g DW), while the highest content of OA (5.75 ± 0.52 mg/g DW) and UA (14.1 ± 0.98 mg/g DW) was determined in leaves of *S. officinalis*. The predominant TA in different tissues of *R. officinalis* was BA, and UA predominated in the studied tissues of *S. officinalis* and *T. persicus*. It has been proposed that the content of secondary metabolites can be significantly affected by genetics^59,60^, the plant species^3,61^ and organ^62,63^, and environmental conditions^64,65^, which is in accordance with the results obtained here. In contrast with the present study, in which BA was the predominant TA in *R. officinalis*, UA was the main TA observed in the study of Razboršek *et al*.^26^, whereas roughly equal proportions of BA, OA and UA were reported by Jäger *et al*.^24^.

The expression levels of the six genes of the TAs metabolic pathway were also determined by qRT-PCR in different plant tissues. The values expressed as means ± SD of three replicates were normalized with *GAPDH* as a reference gene (Fig. 7b). The gene expression levels were compared in two dimensions, cross-gene and cross-tissue. In the cross-gene comparison, the transcript level of a gene was compared with other genes in the same tissue, and in the cross-tissue comparison, the mRNA content of a gene in one tissue was compared with its expression in all other tissues. Significant differences were obtained in the results from the expression level analysis in each tissue and across tissues. Comparison of gene expression across tissues was used to infer the link between elevated expression and metabolite accumulation in tissues. The cross-tissue comparison found a variable expression of the genes in this pathway, with the highest level in leaves, followed by flowers and stems, and the lowest level in roots.

In the case of *SQE*, the same expression pattern was observed in all three plants, being distinctly tissue-specific, and the leaves showing by far the highest level. The expression of this gene was also more pronounced than that of other genes in other plants. The expression levels of *RoSQE*, *SoSQE*, and *TpSQE* were recorded as 5.96 ± 0.18, 4.39 ± 0.48 and 2.19 ± 0.18, respectively. Only in *S. officinalis* was the transcript level of *SoBAS* slightly higher compared to *SoSQE* (4.76 ± 0.45).

The expression level of *OSC* enzymes was consistent with TAs concentration in various tissues of the plants. High levels of expression were recorded for *RoLUS* (4.28 ± 0.24), *SoBAS* (4.76 ± 0.45) and *TpBAS* (1.56 ± 0.25) in leaves. The expression pattern of *SoFDS* varied significantly across the tissues, being strongly expressed in the stem (3.50 ± 0.46) and root (3.18 ± 0.67) and moderately expressed in leaf (2.92 ± 0.49) and flower (2.07 ± 0.32). The expression of the *SQS* gene was observed in all tissues, but the expression pattern of *RoSQS*, *SoSQS*, and *TpSQS* varied according to the tissues.

### Correlation of TAs contents and gene expression

**Figure 8:**
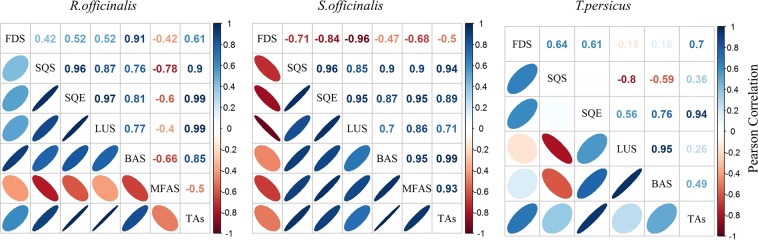
Pearson correlation between the TAs contents and expression of TAs metabolic pathway genes in different tissues of three Lamiaceae species.

The correlation coefficient was calculated to indicate whether the tissue-specific changes in gene expression were directly associated with metabolite accumulation or not (Fig. 8). The amplitude of correlation between the TAs content and gene expression in different plant tissues varied among the plant species, but most genes had a positive correlation with TAs content. All correlations between TAs content and *SQE* gene expression were significant in *R. officinalis*, *S. officinalis*, and *T. persicus* and highly correlated with *RoSQS*, *RoLUS*, *SoSQS*, *SoBAS* and *SoMFAS*, whereas there was low or no correlation with *TpLUS* and *TpSQS* (Fig. 8). Considering the correlation coefficients between the genes of TAs biosynthetic pathway, *SQE* was highly correlated with the *OSC*s, which is dominant in each plant. For example, a strong association was observed between *RoSQE* and *RoLUS*.

## Discussion

SMs are valuable compounds synthesized in plants by a series of chemical and multi-step reactions. In this enzymatic chain, the product of one reaction becomes the substrate for the following one. Therefore, the activity of each enzyme depends on the amount of substrate produced by the previous reaction. Rate-limiting steps in a metabolic pathway require the greatest activation energy and can alter the flux or metabolite concentration in the pathways. The rate of these reactions is influenced by enzyme activity rather than substrate concentration^66^. In this study, gene expression tracking was performed by transcriptome mining, co-expression network analysis, and tissue-specific metabolite-expression analysis to identify the key TAs biosynthetic genes that coordinately regulate the expression of other enzyme-encoding genes in Lamiaceae plants. First, putative key genes were extracted by direct analysis of the structure of the co-expression network. Several characteristics related to the network structure were calculated to provide insights into potential key genes. Highly connected genes in the network reflect relatively high information exchange with other nodes. Thus, this parameter, the relationship with the biological network, is considered as the key property for a gene.

Betweenness centrality (BC), another centrality measure frequently applied in diverse biological research, is defined as the number of shortest paths on which a node lies. “High traffic” nodes are those with large BC values^67^ and a strong impact on the network structure. In the genes with a high value, these two parameters may play an influential role in a given process. In the present study, four genes showed both hubs and nodes with a high betweenness (*MVD1*, *AT3G54250*, *OSC* and *SQE*). In gene co-expression networks, highly connected genes are more effective in the biological pathway. Nevertheless, the results of previous studies indicate that the last three enzymatic steps of the MVA pathway may not be important control points in plant terpene biosynthesis and are not correlated with the rate of terpene formation^68^. A weaker co-expression relationship is observed between this enzyme and downstream genes in the TAs pathway in spite of the high connectivity of *MVD1* in the co-expression network. Thus, it is not a suitable target for metabolic engineering to maximize the yield of TAs.

Furthermore, it is recommended to focus on downstream reactions to understand which genes should be overexpressed to increase TAs production. From the co-expression network, it could be reasonably argued that *SQE* is probably the best choice in this regard, being responsible for increased substrate production or superior channeling of the substrate to the triterpene cyclases. Indeed, *SQE* overexpression has already been introduced as a new strategy for boosting triterpene production in plants^69^. Arabidopsis has six *SQE* copies in its genome^69^. Further, as shown in Fig. 3c, *SQE* has a co-expression relationship with *OSCs*, which points to *OSCs* as regulatory steps in the pathway orienting the biosynthetic flux towards TAs. Since highly co-expressed genes are more likely to be co-regulated, they display prominent connectivity patterns, which may play biologically influential or regulatory roles in a process. However, in several other reports, *FDS* and *SQS* were also co-expressed and used to increase the triterpene production^70–73^.

Metabolic changes are effected mainly through a single or a few enzymes, whose activity is most likely to lead to notable changes in metabolite concentrations. The activity of an enzyme may be related to various factors, including environmental conditions that may cause a change in gene expression. Therefore, the analysis of expression levels, metabolite concentration, and their connections has attracted considerable attention.

As depicted in Fig. 7, the TAs profile varied considerably across the tissues, showing almost the same pattern in the three Lamiaceae plants. The tissue specificity of several metabolic pathway genes was reflected in their expression level. Most of the genes associated with the TAs biosynthetic pathway were expressed mainly in the aerial parts of the plants. None of the studied genes are exclusively expressed in specific tissues, but they exhibited a spatial expression pattern (Fig. 7). Associations between metabolite accumulation and highly expressed genes were obvious in some cases. The most expressed genes in both cross-gene and cross-tissue dimensions are most likely to be involved in metabolite accumulation. For instance, *SQE* and some *OSCs* were highly expressed in leaves and presumably implicated in TAs concentrations, with a good correlation with metabolite accumulation. In contrast, *FDS* and *SQS* were expressed in all the studied tissues, and cross-tissue expression levels may give a narrow description with respect to the TAs concentrations.

The expression pattern of *SoFDS* was almost in line with previously obtained results from *S. guaranitica* and *S. splendens*, which exhibited the highest expression levels in the root^74,75^ and *S. miltiorrhiza*, in the stem^76^. However, our findings differ from those reported for *S. officinalis*, in which young leaves were the site of high expression of *SoFDS*^77^. Additionally, although *FDS* was expressed in all the studied tissues of the Lamiaceae plants, high levels of *SoFDS* were observed in the stem and root, and *RoFDS* and *TpFDS* in the leaf. The Lamiaceae family is rich in bioactive compounds, including phenols, alkaloids, monoterpene, sesquiterpenes, triterpenes, and steroids. Farnesyl diphosphate (FDP), produced by the key enzyme FDS, serves as a precursor for sesquiterpenoids, sterols, triterpenoids, polyprenols, side chains of ubiquinone, and polyisoprenoids such as natural rubber^78,79^. Based on GC-MS analysis of *S. officinalis*, sesquiterpenes formed the main group of compounds found in the stems. Since FDS enzyme activity is closely associated with the accumulation of subsequent products, the high expression level of the *FDS* gene in the stem may be related to the products originating from FDP.

The expression of the *SQS* gene was also observed in all tissues and the expression patterns of *RoSQS*, *SoSQS* and *TpSQS* were exactly matched in the transcriptome analysis of *S. splendens* (SRP126897), with the highest level of transcripts in the leaf  ^75^. In general, 1–3 *SQS* genes are found in plants. In *S. miltiorrhiza*, two copies of *SQS* were reported^80^. Rong *et al*.^81^ confirmed that *SmSQS2* is more expressed in the root than the leaf. Squalene is the precursor for the production of plant sterols as well as triterponoid backbones, so it is important for controlling the flux towards sterol vs. non-sterol products. Both triterpenoids and steroids have been found in the roots of some *Salvia* species^82^. In the plants studied here, a relatively high expression of *SoSQS* was observed in the root.

Although it should be taken into consideration that the nature of transcriptional activity is very dynamic, and can change rapidly with the climatic conditions, developmental stages, and environmental stresses, our findings point to potential key genes for targeting to boost TAs production. In this respect, we evaluated the correlation of specific transcript levels in tissues with BA, OA, and UA accumulation. TAs levels were found to be most significantly correlated with the expression of *SQE* in the three Lamiaceae plants (Fig. 8). In most cases, this gene was also highly correlated with *OSCs*, indicating a co-expression relationship, as observed in the co-expression network analysis. SQE is a rate-limiting enzyme in the biosynthetic pathway, oxidizing squalene into 2,3-oxidosqualene (squalene epoxide) by conducting a stereospecific epoxidation reaction^83^. In addition, it is considered as the biological precursor of all cyclic triterpenoids. Since a rate-limiting enzyme determines the overall rate of a metabolic pathway, SQE has a cascading control of the upregulation of the downstream genes^84^. Therefore, overexpression of this enzyme by metabolic engineering can constitute a system for producing high-value therapeutic TA compounds^85^.

The OSC enzyme family is responsible for a diversifying step in the biosynthesis of various triterpene/sterol scaffolds^56^ by cyclization of 2,3-oxidosqualene. Triterpenes/sterols are derived from the common substrate, 2,3-oxidosqualene, leading to the division of the 2,3-oxidosqualene substrate pool between sterols and a range of triterpenes. TAs scaffolds are derived via a chair–chair–chair (C-C-C) conformation dammarenyl cation by D-ring expansion to form lupeol or further E-ring expansion to form β-amyrin and α-amyrin^86^. These compounds are produced as sole products by the LUS, BAS, and MFAS enzymes, respectively.

OSCs with the same functions exhibit a high sequence identity regardless of plant species. Mutagenesis experiments revealed that the functional diversity of OSCs is determined by certain key amino acid residues, which are essential for the subsequent product specificity of the cyclization process^87^. The end products BA, OA, and UA are produced by further modification by tailoring enzymes (P450s). Additionally, metabolic fluxes can shift the precursor reservoir in the direction of a preferred compound to improve TAs yields. Experimental evidence confirmed that the suppression of a competitive branch may lead to the diversion of the substrate pool toward other OSCs and increase the desired metabolite production^88^. This goal requires the metabolic branch point genes and the upstream genes to be sufficiently flexible to respond to induced genetic and environmental changes such as elicitor application. In the present study, the flexibility of these genes was confirmed by transcriptome mining, which showed that the elicitors YE, MeJA and salicylic acid (SA) caused up-regulation of the *OSCs* or differential transcriptional regulation of each *OSC* in different organs of the plant. The results of various studies confirm that these elicitors can induce *de novo* synthesis of SMs in plants through transcriptional alteration of the genes in the biosynthetic pathway^89^.

Several studies focusing on the regulatory function of FDS and SQS in sesquiterpene, phytosterol, cholesterol, and triterpene saponin biosynthesis^42,70,72,90,91^ report that the activity of these enzymes is associated with the overall yield of these SMs. As illustrated in Fig. 7, the leaves in the studied Lamiaceae plants showed high expression levels of enzymes and metabolite concentrations. In general, striking variability was found among the tissue-specific expression profiles of TAs enzymes. These results indicate that TAs accumulation may be connected with the expression of the genes encoding each of the six enzymes. In addition, the existence of weak or negative correlations between TAs content and the expression of *TpSQS* and *SoFDS* genes does not necessarily mean a negative or no relationship, as in many cases it may be related to a change in the metabolic flow towards non-triterpenic compounds in a specific tissue. Thus, the correlation may be high despite the lower transcript levels in the tissue. Furthermore, the transcriptional level is not always directly related to the enzymatic activity, since several regulatory systems are involved in the availability and activity of the final protein^92^.

Since high-value SMs are produced by plants in low amounts, an in-depth understanding of their biosynthetic machinery is difficult. To increase these compounds, which often have pharmaceutical properties, metabolic engineering necessitates a comprehensive knowledge of metabolic pathways and fluxes, and how these change in response to various types of genetic and environmental perturbations. Other factors that need to be understood include enzyme activity, kinetic properties, post-processing, and all regulatory systems. These factors are critical for elucidating the control of metabolic flux, the desired genetic manipulation, and biotechnological applications.

In the present work, the *in silico* analysis of gene expression and qRT-PCR provided information about the expression behavior of selected genes of the TAs metabolic pathway in the Lamiaceae plants. These results can be considered as a valuable step toward a rational selection of genes suitable for manipulation to improve TAs production. The results of the network analysis were confirmed by the correlation of TAs contents and gene expression. *SQE* and *OSC* expression levels were consistent with the TAs concentration in various plant tissues. An adequate combination of statistical methods and network structure analysis can lead to a significant improvement in our knowledge of the underlying biological functions of a set of genes. Furthermore, this approach can link the structure of the network to a biological process and identify key genes. Further investigation is necessary to identify other factors potentially involved in the regulatory process.

## Methods

### Plant material and data collection

Different plant parts, namely leaf, stem, flower, and root, were collected from three members of the mint family: *Rosmarinus officinalis* L., *Salvia officinalis* L. and *Thymus persicus* (Ronniger ex Rech. f.) Jalas cultivated in the research field of the Medicinal Plants and Drugs Research Institute (MPDRI), Shahid Beheshti University, Evin, Tehran (35° 48′ N, 51° 23′ E at an altitude of 1800 m), Iran. After collection, plant materials were immediately frozen in liquid nitrogen for subsequent metabolic analysis and RNA extraction. A voucher specimen of *T. persicus* (MPH-1673), *S. officinalis* (MPH-2638), and *R. officinalis* (MPH-2639) has been deposited at the herbarium of MPDRI, Shahid Beheshti University (MPH).

Respective datasets were extracted from the Sequence Read Archive (SRA) database of NCBI (https://www.ncbi.nlm.nih.gov/sra/), with accession numbers SRP006813, SRP065934 SRP126897 and, SRP111399. The transcriptome data of SRP006813 included eighteen samples of *R. officinalis*, which were submitted by Michigan State University. Among all the data, six different tissue samples were selected for *in-silico* expression analysis. Assembled transcripts of *R. officinalis* were downloaded from the Medicinal Plant Genomics Resource (http://medicinalplantgenomics.msu.edu/). SRP126897 was associated with the project of RNA-Seq of *S. splendens* (red and purple flowers) with 30 samples of different organs^75^. SRP065934 consisted of eight samples of transcriptional profiles related to *S. miltiorrhiza* cell cultures in response to salicylic acid (SA) elicitation^93^. In addition, SRP111399 was related to five samples of expression profiling by high throughput sequencing of *S. miltiorrhiza* after elicitation by methyl jasmonate (MeJA) and yeast extract (YE)^94^.

### Data preprocessing and differentially expressed gene (DEG) screening

SRA Toolkit version 2.8.2 (https://trace.ncbi.nlm.nih.gov/Traces/sra/sra.cgi) was used to convert the SRA files to FASTQ format. Before any analysis, the quality of raw sequence reads was assessed by FASTQC software. In order to obtain clean reads, Trimmomatic program, Version. 0.36 (http://www.usadellab.org/cms/?page = trimmomatic)^95^ was used to trim low-quality bases from both ends of raw Illumina reads and adapter removal. After trimming, *de novo* assembly was performed using Trinity v2.6.6^96^ at the default KMER setting for the datasets without available assembly files. In the next procedure, the expression levels of the re-constructed transcripts in the individual libraries (different tissues and elicitations) were measured by mapping high-quality filtered reads to assembled transcriptomes using Bowtie-2 via RSEM with recommended RSEM parameters. RSEM uses SAM tool libraries for sorting and indexing the mapping results. “R” and the Bioconductor package edge R were used to analyze the expected counts produced by RSEM. In differential expression analysis of any of the two sample groups, p-values less than 0.05 were considered as the significance threshold and genes with positive and negative log fold change values (logFC) ≥ │2│ were described as up- or downregulated genes, respectively.

### Functional annotation and scrutiny of the expression pattern of the genes involved in TAs biosynthetic pathway

In order to validate and annotate the assembled unigenes, the searches for sequence similarity were performed using the BLAST algorithm against the NCBI non-redundant (nr), SWISS-PROT protein, NCBI nucleotide (nt), and KEGG with the e-value cut-off of 1e-5. In addition, heatmap matrices were used for pattern recognition and visualization to identify expression pattern of the TAs biosynthetic genes in each experiment. Then, all the statistical analysis and plots were conducted in an R environment (version 5.3.0). Further, the co-expression network was constructed in Cytoscape v 3.6.1, an open source software platform, by the GeneMANIA Cytoscape plugin^97^ based on the genes of the TAs biosynthetic pathway. The plugin uses a large database of functional interaction networks from multiple organisms including *Arabidopsis thaliana*. Accordingly, the network properties including specific centrality parameters were calculated by the CentiScaPe2 plugin to identify key nodes and edges in the target biosynthetic pathway.

### Bioinformatics analysis for primer design

The predicted protein sequences of the targeted TAs biosynthetic enzymes in *R. officinalis* were subjected to phylogenetic analysis with the proteins of other plants. The protein sequences of these enzymes were then retrieved from NCBI GenBank databases (http://www.ncbi.nlm.nih.gov/). The amino acid sequences were aligned using ClustalW with default parameters as implemented in the program MEGA7 and the phylogenetic tree was built using the Neighbor-Joining Method. In addition, pairwise genetic distances between the sequences were calculated and the closely related plant species were selected based on the genetic distance matrix for further analysis. Motif-based sequence analysis tools, Multiple Expectation – Maximization for Motif Elicitation (MEME) Suite 5.0.0, (http://meme-suite.org/tools/meme)^98^ were used to discover novel and ungapped motifs in the protein sequences. The parameters of MEME analyses were applied: the distribution of motif occurrences, zero or one per sequence, the number of different motifs (20), minimum motif width (6); and maximum motif width (10). All possible pairs of motifs were detected to design taxa-specific degenerate primers based on conserved regions, and the best primers were selected for the PCR analysis. A Primer-BLAST tool (https://www.ncbi.nlm.nih.gov/tools/primer-blast/) was used to increase the chance of finding specific primer pairs. The tool can incorporate a global alignment mechanism and is designed to be very sensitive in detecting potential amplification targets. The properties of each primer such as melting temperature, GC content percentage, and polymerase chain reaction (PCR) suitability were checked with some online tools like OligoCalc (http://biotools.nubic.northwestern.edu/OligoCalc.html) and OligoAnalyzer (https://eu.idtdna.com/calc/analyzer).

### RNA isolation, RT reaction, PCR amplification and sequencing

Total RNA was extracted using Trizol (TRI reagent Sigma-Aldrich, USA), and the first-strand cDNA was synthesized from 2 µg of the isolated RNA templates by reverse transcriptase with Oligo-(dT)18 primers according to the instructions of the RevertAidTM H M-MuLV First-Strand cDNA Synthesis Kit (Thermo Fisher Scientific, Fermentas). PCR was performed using the cDNA prepared from the leaves of the studied plants as templates to amplify DNA fragments encoding TAs biosynthetic enzymes. Each PCR reaction was set up in 25 μl volume containing 12.5 μl 2X PCR master mix (Sinaclon, Iran), 50 ng of the synthesized cDNA and gene-specific primers (Table 1). The PCR conditions included initial denaturation at 94 °C for 5 min, followed by 35 cycles [94 °C for 1 min, 50–55 °C (depending on the primer combination) for 30 seconds, 72 °C for 30 sec] and a final extension at 72 °C for 5 min. The amplified products were separated on 1.5% agarose gel and visualized by ethidium bromide staining. Purification and concentration of the PCR product were carried out using the GF-1 Gel DNA Recovery Kit (Vivantis). Purified PCR products were sequenced and screened in the GenBank (NCBI) database for matching homology. First, the amplicon sequences were aligned by applying BLASTx against protein databases, including the non-redundant (Nr) protein database, Swiss-Prot (http://www.expasy.ch/sprot/), and Kyoto Encyclopedia of Genes and Genomes (KEGG) (http://www.genome.jp/kegg/), and then using BLASTn against the Nucleotide (Nt) database (with a threshold of E < 1.0E-5).

### Transcript levels of the genes involved in the biosynthesis of TAs

qPCR was done to investigate the transcript levels of six genes encoding TAs biosynthetic enzymes in different organs of *R. officinalis*, *S. officinalis* and *T. persicus*. The qPCR reactions were performed in a 20 μl volume including 10 μl qPCR GreenMaster (Jena Bioscience, Germany), 50 ng of cDNA and 300 nM of each primer in a Rotor-Gene® 6000 (Qiagen, Germany). PCR amplification was performed with three technical replications under the following conditions: 2 min at 95 °C, followed by 45 cycles of 95 °C for 20 s, 53 °C for 20 s, and 72 °C for 20 s. Then, glyceraldehyde-3-phosphate dehydrogenase (*GAPDH*) was used to normalize the real-time PCR data as an internal control. The expression level was calculated according to the 2^−ΔΔCt^ method (Livak and Schmittgen, 2001) based on the mean of three independent determinations of the threshold cycle. In addition, the value of 2^−ΔΔCt^ was used to represent the relative expression of each gene.

### Extraction and HPLC analysis

In the next procedure, reverse phase-HPLC was used to determine the content of BA, OA, and UA in the studied plant parts. As mentioned, lyophilized and powdered plant material (1.0 g) was drenched in MeOH (40 mL) and immediately sonicated at 30% amplitude for 40 min at room temperature^99,100^. The analysis was conducted using a Water symmetry C_18_ column with methanol: phosphoric acid: water (87:0.05:12.95) as an isocratic elution mode and UV detection (λ = 210 nm). All standards of BA, OA, and UA were purchased from Sigma-Aldrich and the standard solutions were prepared by serial dilutions of stock solutions at a concentration range of 50–800 ppm. A calibration curve (standard curve) was generated by injecting the series of calibration standards and plotting concentration against the peak area in Excel 2016. Then, the three TAs in the unknown samples were quantified by the standard equation obtained from the calibration curve, and the correlation coefficients (R^2^) were calculated for each of the three compounds. Each sample was run three times to evaluate the precision.

### Statistical analysis

All data are expressed as means ± SD. Normality was assessed using the Shapiro-Wilk test and the data were analyzed by univariate one-way ANOVA. Duncan’s multiple range test was used for post hoc analysis. To measure the intensity of correlation between the expression of each gene and TAs content, the Pearson correlation coefficient was calculated for different organs of each plant and represented in correlograms. Analyses were performed by R statistical software v.3.5.0 (freely available at http://www.r-project.org). In all the analyses, the P < 0.05 was used as the significance threshold.
